# *Ferrodraco lentoni* gen. et sp. nov., a new ornithocheirid pterosaur from the Winton Formation (Cenomanian–lower Turonian) of Queensland, Australia

**DOI:** 10.1038/s41598-019-49789-4

**Published:** 2019-10-03

**Authors:** Adele H. Pentland, Stephen F. Poropat, Travis R. Tischler, Trish Sloan, Robert A. Elliott, Harry A. Elliott, Judy A. Elliott, David A. Elliott

**Affiliations:** 1 Faculty of Science, Engineering and Technology, Swinburne University of Technology, John St, Hawthorn, Victoria, 3122 Australia; 2 Australian Age of Dinosaurs Natural History Museum, The Jump-Up, Winton, Queensland, 4735 Australia

**Keywords:** Taxonomy, Phylogenetics, Palaeontology, Palaeontology, Taxonomy

## Abstract

The Australian pterosaur record is poor by world standards, comprising fewer than 20 fragmentary specimens. Herein, we describe the new genus and species *Ferrodraco lentoni* gen. et sp. nov., based on the most complete pterosaur specimen ever found in Australia, and the first reported from the Winton Formation (Cenomanian–lower Turonian). The presence of premaxillary and mandibular crests, and spike-shaped teeth with subcircular bases, enable *Ferrodraco* to be referred to Anhangueria. *Ferrodraco* can be distinguished from all other anhanguerian pterosaurs based on two dental characters: the first premaxillary and mandibular tooth pairs are small; and the fourth–seventh tooth pairs are smaller than the third and eighth ones. *Ferrodraco* was included in a phylogenetic analysis of Pterosauria and resolved as the sister taxon to *Mythunga camara* (upper Albian Toolebuc Formation, Australia), with that clade occupying the most derived position within Ornithocheiridae. *Ornithocheirus simus* (Albian Cambridge Greensand, England), *Coloborhynchus clavirostris* (Valanginian Hastings Sands, England), and *Tropeognathus mesembrinus* (upper Aptian–lower Albian Romualdo Formation, Brazil) were resolved as successive sister taxa, which suggests that ornithocheirids were cosmopolitan during the Albian–Cenomanian. Furthermore, the stratigraphic age of *Ferrodraco lentoni* (Cenomanian–lower Turonian) implies that anhanguerians might have survived later in Australia than elsewhere.

## Introduction

Pterosaurs are known from Mesozoic strata on every continent, with their fossil record spanning the Late Triassic–end Cretaceous^1^. Despite this, pterosaur remains are typically incomplete and fragmentary, primarily because their bones have thin cortices and are typically hollow^2^. Pterosaurs are rare in Australia, with their fossil record restricted to the Cretaceous and comprising isolated and fragmentary bones from Queensland^3–9^, New South Wales^10^, Western Australia^11,12^ and Victoria^13,14^. The Victorian pterosaur fossils remain undescribed^14^, whereas those from New South Wales can be identified no more precisely than Anhangueria indet^10^. By contrast, the few pterosaur fossils from Western Australia evidently represent an anhanguerian^12^ and an azhdarchid^11,15^.

All of the pterosaur remains known from Queensland derive from the Eromanga Basin, all but one are from the upper Albian Toolebuc Formation (the exception being a ctenochasmatoid from the upper Albian Mackunda Formation^6^), and most have been tentatively referred to the Ornithocheiridae *sensu* Unwin^16,17^. Although some authors (e.g. Unwin^16,17^) have considered the Anhangueridae as a junior synonym of the Ornithocheiridae, a recent phylogenetic analysis by Andres and Myers^18^ resolved Anhangueridae and Ornithocheiridae as sister taxa. Although it is possible that some of the Queensland ‘ornithocheirids’, particularly those identified as cf. *Anhanguera*^6^, might in fact be anhanguerids, this cannot be determined because of their incomplete preservation. The only named pterosaur species from Australia — *Mythunga camara* (Queensland Museum [QM, Brisbane, Queensland, Australia] F18896)^5,9^ and *Aussiedraco molnari* (QM F10613)^3,8^ — are from the upper Lower Cretaceous (upper Albian) Toolebuc Formation of Queensland. *Mythunga* is represented by an incomplete skull found near Hughenden, comprising incomplete premaxillae, maxillae, dentaries, and other cranial elements preserved in matrix within the nasoantorbital fenestra, and was originally interpreted as a short-snouted archaeopterodactyloid^5^. A recent redescription of this taxon demonstrated that it was not short-snouted^9^; moreover, the inclusion of *Mythunga* in a phylogenetic analysis (based on a modified version of the Andres *et al*.^19^ dataset) suggested that it was an anhanguerian, not an archaeopterodactyloid. However, the inclusion of *Mythunga* in this analysis reduced the resolution within Anhangueria to such a degree that its position within that clade could not be established^9^. By contrast, *Aussiedraco* is represented by a mandibular symphysis found near Boulia that was initially assigned to aff. *Ornithocheirus*^3^ but later classified more broadly as a member of Pteranodontoidea^8^.

Here we report on, and briefly describe, a new anhanguerian pterosaur species from the Cenomanian–lower Turonian (lower Upper Cretaceous) Winton Formation of Queensland, northeast Australia. This specimen — discovered in April 2017 by one of the authors (RAE) — is the first pterosaur reported from the Winton Formation, and is also the most complete pterosaur ever found in Australia.

## Results

### Systematic palaeontology

Pterosauria Kaup, 1834

Pterodactyloidea Plieninger, 1901

Ornithocheiroidea Seeley, 1891 *sensu* Kellner, 2003

Anhangueria Rodrigues and Kellner, 2013

Ornithocheirae Seeley, 1870

Ornithocheiridae Seeley, 1870

Ornithocheirinae Andres, Clark and Xu, 2014

***Ferrodraco lentoni*** gen. et sp. nov.

### Etymology

From the Latin *ferrum* (iron), in reference to the ironstone preservation of the holotype specimen, and the Latin *draco* (dragon). The species name honours former Winton Shire mayor Graham Thomas ‘Butch’ Lenton, in recognition of his years of service to the Winton community and support to the Australian Age of Dinosaurs Natural History Museum.

### Holotype

Australian Age of Dinosaurs Fossil (AODF, Winton, Queensland, Australia) 876 (‘Butch’): anterior portion of skull comprising partial premaxillae, maxillae and dentaries (including premaxillary and mandibular crests and the mandibular symphysis); partial left frontal; left mandibular articular region comprising the surangular, angular and articular; five partial cervical vertebrae; partial right scapulocoracoid; partial left ulna; partial left radius; left proximal and distal carpals; left metacarpal IV; proximal end of right metacarpal IV; fragmentary left non-wing manual phalanges; partial left first wing phalanx (IV-1); and associated fragments. Several elements, including the skull and mandible and many of the appendicular elements (based on key-fits between adherent matrix on anatomically adjacent elements) were clearly articulated post-fossilisation; however, erosion and soil rotation led to fragmentation of the specimen prior to its excavation.

### Type horizon and locality

**Figure 1:**
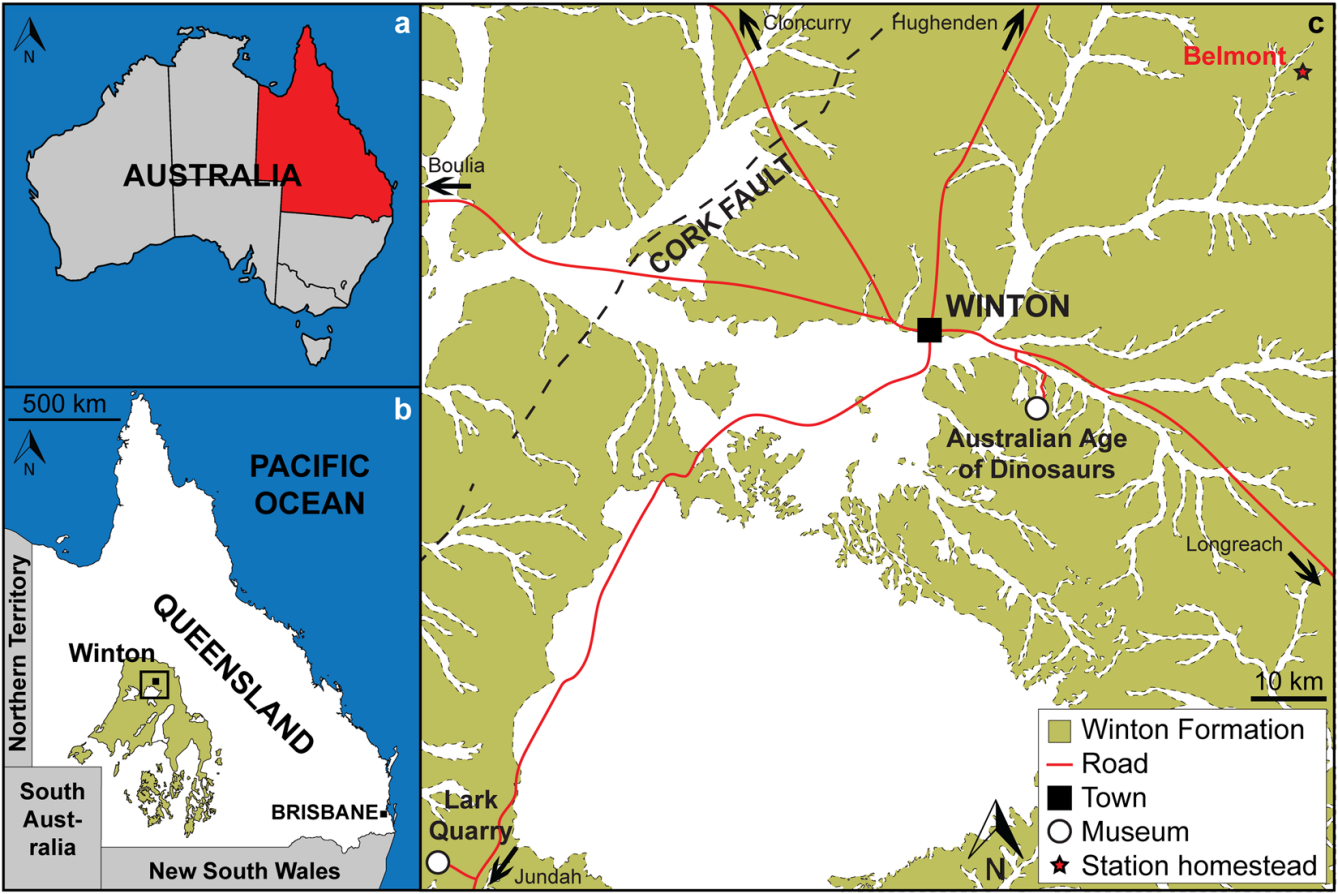
Location of the *Ferrodraco lentoni* gen. et sp. nov. type locality (AODL 245). (**a**) Map of Australia showing the location of Queensland (modified from Poropat *et al*.^60^). (**b**) Map of Queensland showing the distribution of Winton Formation outcrop (modified from Poropat *et al*.^60^). (**c**) Map of the Winton area showing Winton Formation outcrop, the location of Belmont Station, and museums in the region. This map was drafted by S.F.P. in Adobe Illustrator CC 2017, and incorporates geological information from Vine^61^ and Vine & Casey^62^ [© Commonwealth of Australia (Geoscience Australia) 2019. This product is released under the Creative Commons Attribution 4.0 International Licence. http://creativecommons.org/licenses/by/4.0/legalcode].

Winton Formation (Cenomanian–lower Turonian^20,21^); Australian Age of Dinosaurs Locality (AODL, Winton, Queensland, Australia) 245 (the ‘Pterosaur Site’), Belmont Station, Winton, Queensland, Australia (Fig. 1).

### Diagnosis

Anhanguerian pterodactyloid diagnosed by the following autapomorphies: (1) first tooth pair of the premaxilla and mandible smaller than other anterior teeth; (2) fourth up to seventh teeth smaller than third and eighth.

### Description

**Figure 2:**
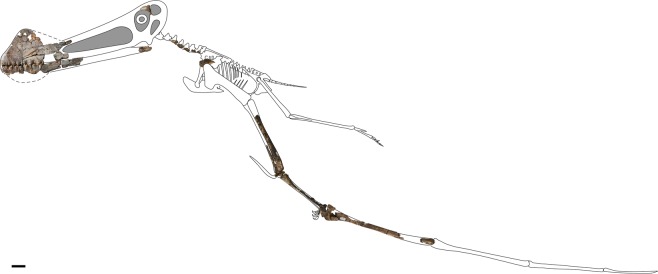
*Ferrodraco lentoni* gen. et sp. nov. holotype specimen AODF 876. All preserved elements were photographed and scaled to the same size, then articulated where possible. These were then used as the basis for the scaling of the skeletal reconstruction, the missing parts of which were based on the skeletal reconstruction of *Tropeognathus mesembrinus* by Witton^63^. Scale bar = 50 mm.

The holotype specimen of *Ferrodraco lentoni* (Fig. 2) is three-dimensionally preserved, with some elements crushed and distorted (e.g. ulna, radius, left metacarpal IV, manual phalanx IV-1). The fused scapulocoracoid and ossified extensor tendon process of wing phalanx IV-1 indicate that the type individual was ontogenetically mature; however, CT scans of the carpus demonstrate that the proximal and distal carpals were not sutured. Based on measurements of the type specimen (Table 1), and comparisons with other anhanguerian pterosaurs, the wingspan of *Ferrodraco* was approximately 4 m (see summary in Pentland and Poropat^9^).

**Table 1:** Measurements of *Ferrodraco lentoni* (AODF 876) in millimetres. Measurements based on incompletely preserved elements are indicated with an asterisk (*).

Element		Anteroposterior length		Transverse width		Dorsoventral height
Upper jaw		270*		20*		140*
Frontal		40*		19*		38*
Lower jaw		300*		33*		32*
Mandibular symphysis		180*		22*		35*
Surangular, angular and articular		50*		23*		20*
Cervical vertebra A		27*		18*		18*
Cervical vertebra B		28*		19*		9*
Cervical vertebra C		21*		11*		21*
Cervical vertebra D		16*		15*		7*
Cervical vertebra E		13*		14*		5*
**Measurement**	**Proximodistal length**	**Maximum width**	**Dorsoventral height**	**Proximodistal length**	**Maximum width**	**Dorsoventral height**
**Element**	**Left**			**Right**		
Scapulocoracoid	—	—	—	52*	37*	—
Ulna	235*	57	—	—	—	—
Radius	110*	24	—	—	—	—
Carpus	23	55	—	—	—	—
Metacarpal IV	205	37*	19*	100*	45	27
Manual phalanx IV—1 (proximal)	310*	54*	—	—	—	—
Manual phalanx IV-1 (distal)	52*	22*	—	—	—	—

Assignment of *Ferrodraco* to the Anhangueria is supported by several cranial synapomorphies, including the presence of a mandibular groove, smooth and blade-like premaxillary and mandibular crests, and spike-shaped teeth^22,23^. *Ferrodraco lentoni* can be distinguished from other anhanguerians by the following combination of characters: anterior margin of the premaxilla flattened and triangular, first tooth pair of the premaxilla projecting vertically and slightly elevated relative to jawline; anterior portions of jaws not laterally expanded; vertically oriented teeth decreasing in size posteriorly; alveolar borders inflated relative to jawline; tall premaxillary crest level with anterior margin of skull, rises steeply at an angle of 60° with rounded dorsal margin.

**Figure 3:**
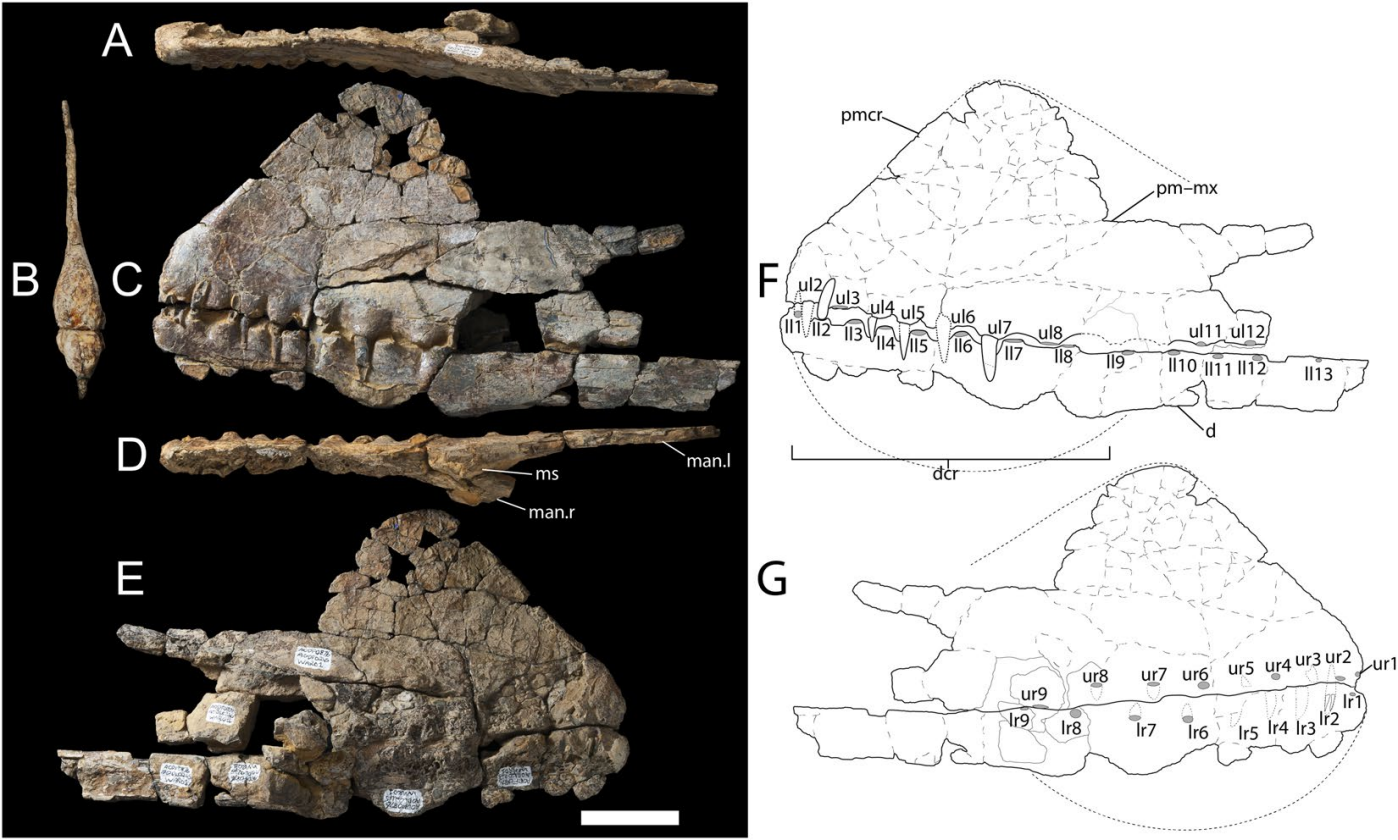
*Ferrodraco lentoni* gen. et sp. nov. holotype skull and mandible AODF 876. (**A**) dorsal view; (**B**) anterior view; (**C**) left lateral view; (**D**) ventral view; (**E**) right lateral view; (**F**) schematic of left lateral view; and (**G**) schematic of right lateral view. Abbreviations: d, dentary; dcr, (preserved base of) dentary crest; ll#, lower left (alveolus number); lr#, lower right (alveolus number); man, mandibular ramus; ms, mandibular symphysis; pmcr, premaxillary crest; pmx-mx, premaxilla–maxilla; ul#, upper left (alveolus number); ur#, upper right (alveolus number). Scale bar = 50 mm.

**Figure 4:**
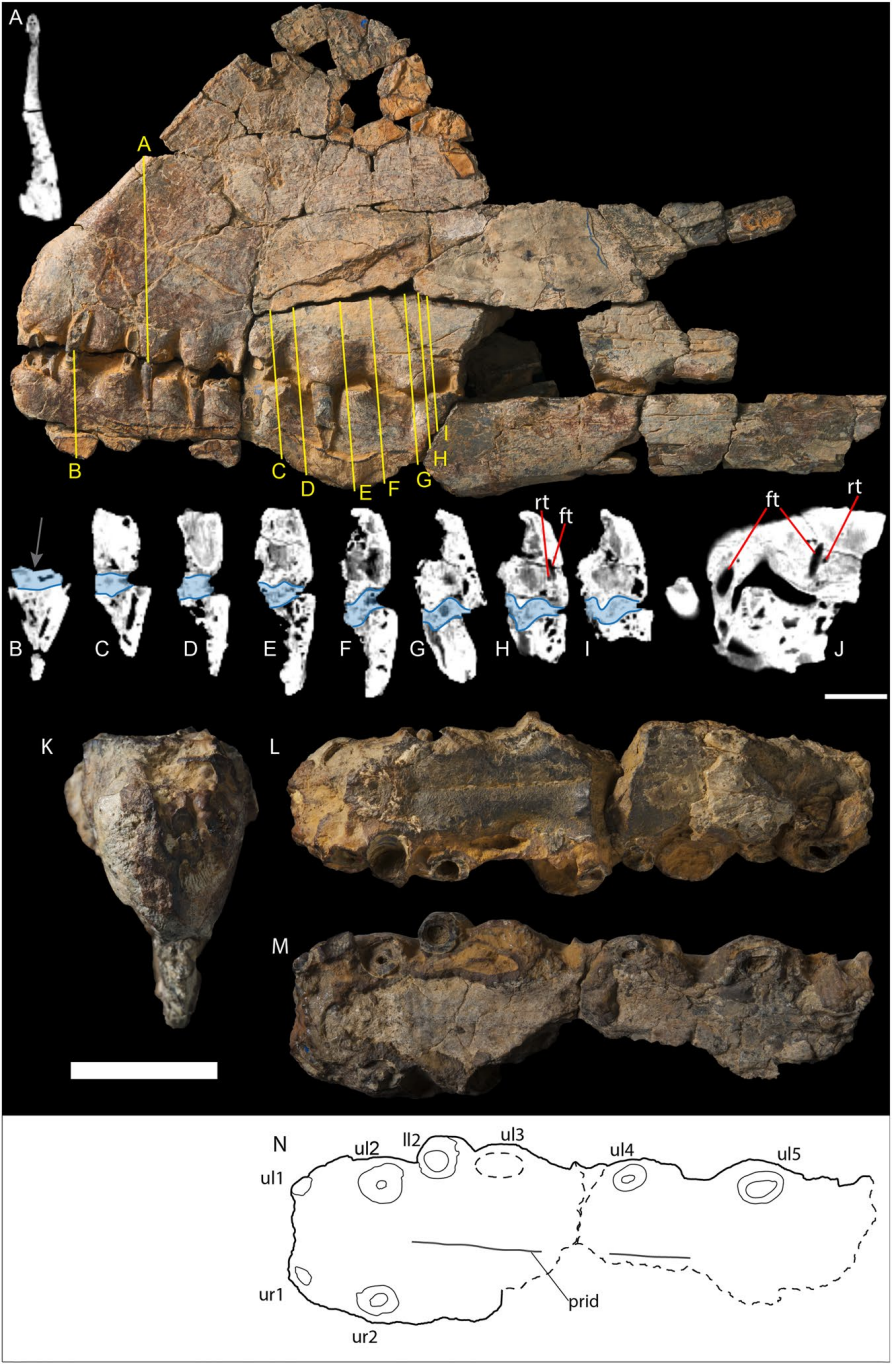
*Ferrodraco lentoni* gen. et sp. nov. holotype rostral sections AODF 876. Cross-section (**A**) internal structure of premaxillary crest; (**B**) weak mandibular groove (grey arrow) and internal structure indicates the mandible is incomplete ventrally; (**C**–**I**) demonstrate variation in the depth of the palatal ridge and corresponding mandibular groove, with ironstone matrix represented by shaded region; (**H**) demonstrates replacement tooth is located lingual to the functional tooth; (**J**) section through (**H**) demonstrating the replacement tooth is distal to the functional tooth. The location of the functional and replacement teeth was based on their pulp cavities (black). Co-ossified dentaries in (**K**) anterior and (**L**) dorsal (occlusal) views. (**M**) Co-ossified premaxillae and maxillae in ventral (occlusal) view. (**N**) Schematic of premaxillae and maxillae in ventral (occlusal) view showing the subtle longitudinal palatal ridge. Abbreviations: ft, functional tooth; ll#, lower left; prid, palatal ridge; rt, replacement tooth; ul#, upper left; ur#, upper right. Scale bars: 20 mm for all figures.

The premaxilla–maxilla, as preserved, is anteroposteriorly longer than it is dorsoventrally tall (Fig. 3; Table 1), with a transversely thin premaxillary crest (4 mm; Fig. 3). This comprises two thin lateral plates separated by trabeculae, as indicated by CT scans (Fig. 4A). The skull is not laterally expanded, as in other anhanguerian pterosaurs^23^. The premaxillary crest is incomplete posteriorly; however, the anterior margin is complete and confluent with the anterior margin of the skull, despite being inclined posterodorsally (Fig. 3C). The premaxillary crest is comparatively large, with a high and blunt anterior margin, as in *Coloborhynchus clavirostris*^24^ and *Siroccopteryx moroccensis*^25^. However, *Ferrodraco* differs from *Coloborhynchus clavirostris* because its first premaxillary teeth are smaller, vertically oriented, and more ventrally positioned. The premaxillary crest of *Ferrodraco* is dorsoventrally taller than that of *Siroccopteryx*^25^. The transverse width of the premaxillary crest in *Ferrodraco* is 2.5 mm, similar to that of *Tropeognathus mesembrinus*^26^ but divergent from the condition in *Ornithocheirus simus*^27^ wherein the crest is transversely wider. The crest of *Ferrodraco*, although broadly similar to that of *Tropeognathus mesembrinus*, differs in that it is less rounded overall, and in that its anterior margin slopes posterodorsally at an angle of 60° rather than ~70° (estimated based on published schematic in Wellnhofer^26^). As preserved, the premaxillary crest of *Ferrodraco* has an anteroposterior length of 131 mm and reaches its maximum dorsoventral height (128 mm, as measured from the ventral margin of the seventh alveolar rim) 99 mm posterior to the anterior margin of the skull, directly above the seventh upper alveolus.

An anteroposteriorly elongate, transversely narrow palatal ridge, which occludes with the mandibular groove (observed in CT data), initiates posterior to the second pair of alveoli (Fig. 4B). The apparent longitudinal groove visible on the mandible in dorsal view (Fig. 4L) is an impression of the palatal ridge in adherent ironstone matrix. Concomitant with the palatal ridge, this groove (again, based on CT data) became deeper and more pronounced posteriorly (Fig. 4B–I), only terminating at the posterior margin of the mandibular symphysis (Fig. 4I), as in *Tropeognathus mesembrinus*^26^. The mandibular symphysis, formed by the complete coalescence of the dentaries anteriorly, is 180 mm long anteroposteriorly. This is significantly shorter than that of *Anhanguera piscator* (259 mm)^28^ and of the similarly sized specimen Staatliche Naturwissenschaftliche Sammlungen Bayerns–Bayerische Staatssammlung für Paläontologie und Geologie (SNSB–BSPG, Munich, Germany) 1987 I 47 (“*Anhanguera robustus*”)^28^. Although no precise length measurement for the mandibular symphysis of *Tropeognathus mesembrinus* has been published, Wellnhofer^26^ stated that it almost equal to one-third the total dentary length in that taxon (520 mm; so ~173 mm) — absolutely shorter than in *Ferrodraco*. The preserved portions of the diverging mandibular rami in *Ferrodraco* were relatively straight, although their morphology further posteriorly cannot be determined. The symphysis is not laterally expanded.

The anterior margin of the mandible is rounded in lateral view. The ventral margin of the anteriormost portion of the mandibular symphysis is convex, but is also mostly missing on the right side. Nevertheless, the preserved portion of the left dentary forms a laterally compressed plate of bone that is inclined ventromedially (Fig. 4B), and is interpreted here as evidence for a mandibular crest. CT scan data demonstrate that the internal texture of the dentary at its incomplete ventral margin is similar to that of the premaxillae. Furthermore, the anteroventral dip of the ventral margin of the dentaries, anterior to the mandibular symphysis, supports the existence of a mandibular crest. Given that it clearly did not extend posterior to the mandibular symphysis, the maximum length of this crest was 126 mm, whereas it was clearly taller dorsoventrally than the tallest preserved portion (10 mm). The preserved portion of the left dentary hosts thirteen alveoli, whereas nine alveoli are present on the preserved portion of the right one (Fig. 3F,G).

The left premaxilla–maxilla preserves the anteriormost eight consecutive alveoli and two additional more mesial alveoli interpreted as 11 and 12 based on interalveolar spacing and the number of teeth in the left dentary, which preserves thirteen alveoli in total (Fig. 3F). By contrast, the right premaxilla–maxilla preserves the nine anteriormost consecutive alveoli (Fig. 3G). The first pair of premaxillary teeth is positioned slightly dorsal to the rest of the tooth row, as in *Tropeognathus mesembrinus*^26^. Isolated jaw fragments preserving vacant alveoli were also recovered from the site, but their absolute positions within the jaws cannot be determined.

The lateral surfaces of the premaxillae–maxillae and dentaries show undulation, as a result of the inflation of the alveolar borders relative to the jawline. The degree of alveolar border inflation decreases posteriorly from the eighth alveolus on the left dentary. Based on their alveolar diameter, the maxillary teeth are subequal in size to, if not slightly larger than, the dentary teeth. In both the premaxillae and dentaries, the first tooth pair is situated on the anteriormost margin.

More than 40 isolated teeth and tooth fragments are present in the *Ferrodraco lentoni* holotype (see Supplementary Information) and, in some cases, their corresponding alveoli have been identified. The teeth are spike-shaped, vertically oriented and slightly lingually recurved, with the displacement of tooth curvature less than the tooth diameter. The transverse cross-sectional shape of the base of each tooth is oval, with each being longer mesiodistally than wide labiolingually, and the crown height of each tooth being less than four times its basal width. The spacing between successive teeth is greater than the diameter of either of those teeth, and the mesiodistal length of the interalveolar spaces increases posteriorly. CT scan data demonstrate that *Ferrodraco* had a distolingual pattern of tooth replacement (Fig. 4H–J), lending further support to the hypothesis that this mode of replacement was typical for dentulous pterosaurs^29^.

**Figure 5:**
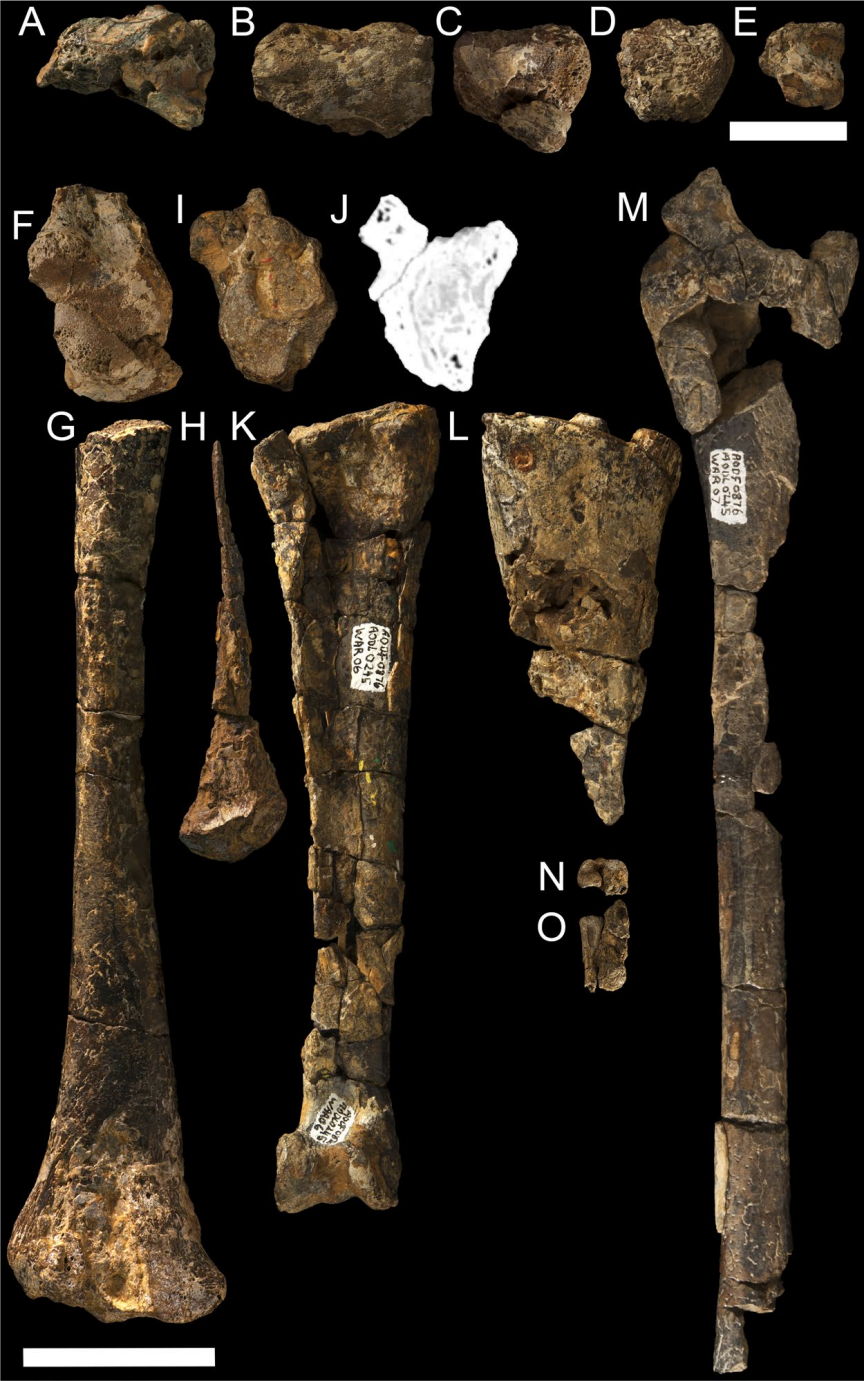
*Ferrodraco lentoni* gen. et sp. nov. holotype postcranial material AODF 876. (**A**–**E**) Cervical vertebrae A–E (all in ventral view); (**F**) Right scapulocoracoid (lateral view); (**G**) Left ulna preserving distal end (anterior view); (**H**) Left radius preserving distal end (posterior view); (**I**) Left syncarpus (distal view); (**J**) Internal structure of left syncarpus (ventral view), showing that the proximal (left) and distal (right) carpals were preserved together but were not fused (contact line indicated by red arrow); (**K**) Left metacarpal IV (posterior view); (**L**) Proximal end of right metacarpal IV (posterior view); (**M**) Proximal end of left wing phalanx I (dorsal view); (**N**,**O**) Proximal end of non-wing manual phalanx (with adherent matrix) in proximal (**N**) and (**O**) ventral views). Scale bars: A–E = 20 mm, F–O = 50 mm.

Five partial cervical vertebrae of *Ferrodraco* are preserved (Fig. 5A–E); however, their respective positions within the cervical series cannot be determined because of their fragmentary state. The right scapulocoracoid of *Ferrodraco* is represented only by part of the glenoid fossa, including the supraglenoidal buttress, the articular surface and the lower tubercle (Fig. 5F). The left ulna is incompletely preserved, being represented by most of the shaft and the distal end (Fig. 5G). The dorsal half of the distal articular surface of the ulna is saddle-shaped (concave dorsoventrally, convex anteroposteriorly), and the dorsal margin is a sharply-defined ridge. Centrally, the ulna bears a weakly developed tuberculum, and ventral to this the fovea carpalis is present. Only the distal portion of the left radius of *Ferrodraco* is preserved (Fig. 5H). The distal articular surface of the radius is incomplete but was clearly suboval in outline, as in other anhanguerians^28,30^, and dorsoventrally expanded. Although the preserved portion of the radius shaft is crushed and distorted, its diameter was clearly less than half that of the ulna.

The left syncarpus is incomplete, and represented by the proximal and distal carpals preserved together but not fused; the lateral carpal is not preserved (Fig. 5I,J). The distal and proximal articular surfaces of the carpus are subequal in size, with the distal carpal triangular in outline. Both left and right fourth metacarpals are present in the holotype of *Ferrodraco* (Fig. 5J,K). Although only the proximal portion of right metacarpal IV has been recovered, it is better preserved than left metacarpal IV, which is complete but anteroposteriorly flattened. The proximal end of left metacarpal IV is less robust than the right, although this is clearly a result of distortion. Given that the right element is regarded as being more representative of the original morphology of metacarpal IV, it is clear that the proximal articular surface of this element was subrectangular in outline. The left metacarpal IV of *Ferrodraco* is proximodistally shorter than those of *Anhanguera piscator* (256 mm)^28^, *Arthurdactylus conandoylei* (227 mm)^31^, Staatliches Museum für Naturkunde Karlsruhe (SMNK, Karlsruhe, Germany) 1136 PAL (254 mm)^31^, and Museums Victoria (NMV, Melbourne, Australia) P197962 (212 mm)^7^. By contrast, it is proximodistally longer than those of *Boreopterus cuiae* (94 mm)^32^, SMNK 1134 PAL (165 mm)^31^ and SMNK 1135 PAL (175 mm)^31^. Despite the poor preservation of left metacarpal IV, it is clear that the dorsal and ventral distal condyles were separated by a median ridge. Only fragments of digits I–III and the proximal end of an indeterminate manual phalanx found in matrix has been recovered (Fig. 5M,N). Preliminary observation of the latter suggests that another, less well-preserved manual phalanx is also preserved. Although part of right metacarpal IV was recovered, the preserved portions of digits I–III are most likely from the left side, given that they were found in close proximity to left metacarpal IV. The manual phalanges are round to subtriangular in transverse cross-section. The left first wing phalanx is represented by the proximal portion and shaft (Fig. 5L), with the proximal articular surface (Table 1) dorsoventrally shorter than that of *Anhanguera piscator* (65 mm)^28^. The extensor tendon process also bears an oval pneumatic foramen, which is visible in ventral view.

### Comparisons with australian pterosaurs

#### Comparisons between *Ferrodraco* and *Mythunga*

**Figure 6:**
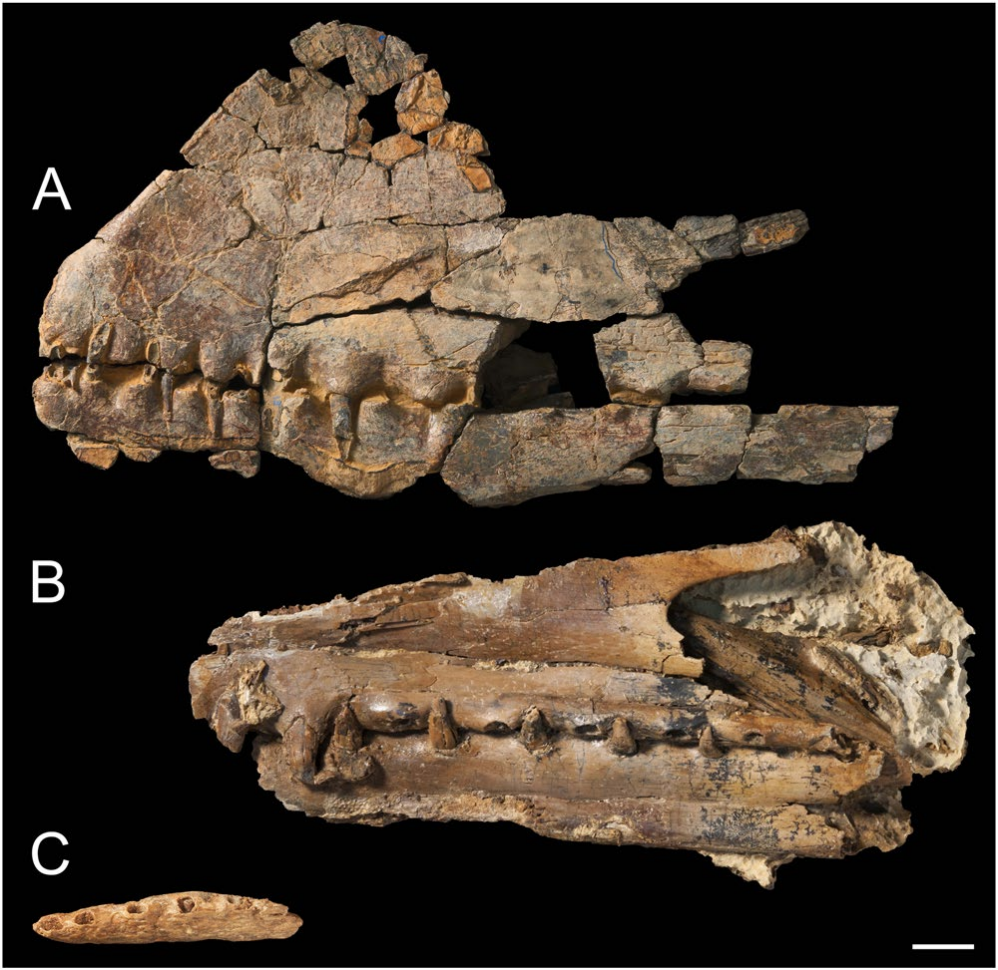
Australian pterosaur holotype cranial material. (**A**) *Ferrodraco lentoni* gen. et sp. nov. holotype skull and mandible (AODF 876); (**B**) *Mythunga camara* Molnar and Thulborn^5^ holotype skull and mandible (QM F18896); and (**C**) *Aussiedraco molnari* Kellner, Rodrigues and Costa^8^ holotype mandible (QM F10613). Scale bar = 20 mm.

Given that only the anterior third of the skull and mandible (Fig. 6A), and the posteriormost portion of the left mandible, of *Ferrodraco* is preserved, there is little anatomical overlap between it and the holotype specimen of *Mythunga camara*^5,9^, which preserves only the mid-section (Fig. 6B). The CT scan data did not unequivocally demonstrate that any sections of the nasoantorbital fenestra margin were preserved in *Ferrodraco*; thus, the only anatomical landmark preserved in both it and *Mythunga* was the mandibular symphysis. Unfortunately, the position of the mandibular symphysis relative to the anterior margin of the nasoantorbital fenestra shows significant variation among anhanguerians^9^. Consequently, we restricted our comparisons between *Ferrodraco* and *Mythunga* to the teeth and alveoli.

The teeth of *Mythunga* and *Ferrodraco* are similar in that they are spike-shaped and labiolingually recurved; however, the teeth of *Mythunga* are much more robust, with the height to mesiodistal width ratio of complete teeth varying between 1.3 and 2.0 (see Table 2 in Pentland and Poropat^9^). By contrast, in *Ferrodraco* this ratio varies between 2.3 and 7.5 (see Supplementary Information). The fact that the teeth preserved are from the mid-section has probably impacted the degree of variation observed in this ratio, given that anterior teeth are larger than the posterior teeth in members of this clade. It is therefore likely that the anterior teeth of *Mythunga* would have been even larger and more robust than in *Ferrodraco*.

*Ferrodraco* and *Mythunga* are similar in that both have raised alveolar borders; however, these appear to be more prominent in the anteriormost portion of the *Mythunga* holotype. Unlike *Mythunga*, *Ferrodraco* does not appear to possess nutrient foramina in association with its alveolar borders, although this might be an artefact of preservation. CT scan data did not unequivocally demonstrate nutrient foramina were preserved in association with alveolar borders in *Ferrodraco*. Although the total tooth count cannot be determined for either *Ferrodraco* or *Mythunga*, provisional comparisons can be made based on the interalveolar spacing observed posterior to the mandibular symphysis. The interalveolar spacing increases posterior to the mandibular symphysis in *Ferrodraco*, whereas in *Mythunga* it does not decrease until four tooth positions posterior to the mandibular symphysis (see Table 3 in Pentland and Poropat^9^). These differences in interalveolar spacing and dentition imply that both tooth count and arrangement were different in these taxa.

#### Comparisons between *Ferrodraco* and *Aussiedraco*

The most striking difference between *Aussiedraco* (Fig. 6C) and *Ferrodraco* is that the latter possesses a mandibular crest; consequently, the mandible of *Ferrodraco* is relatively and absolutely taller dorsoventrally than that of *Aussiedraco*, despite the incomplete preservation of its ventral margin. However, given that some pterosaurs were evidently sexually dimorphic, with the most obvious manifestation of this being the development (or not) of the cranial crest(s)^33^, this character alone is insufficient to distinguish *Ferrodraco* from *Aussiedraco*.

The mandible of *Ferrodraco* is more distally expanded and more robust than that of *Aussiedraco*. Furthermore, the left lateral surface of the mandible of *Ferrodraco* is slightly convex, whereas that of *Aussiedraco* is relatively straight. The occlusal surface of the mandibular symphysis in *Ferrodraco* is straight in lateral view, whereas in *Aussiedraco* it is markedly convex. In *Aussiedraco*, the anterior portion of the mandibular groove is more prominent than that of *Ferrodraco*, and appears most pronounced from the posterior margin of the third alveolus until the posterior margin of the fourth alveolus in the former taxon. However, in *Ferrodraco* the mandibular groove extends from the posterior margin of the 2^nd^ tooth locus to the mandibular symphysis, which might not have been the case in *Aussiedraco*.

*Aussiedraco* differs from *Ferrodraco* (and *Mythunga*) in that its alveolar borders are not raised. As described by Kellner *et al*.^8^, the first four alveoli of *Aussiedraco* are oriented slightly more dorsolaterally than the fifth alveolus, which faces more dorsally. By contrast, all dentary alveoli anterior to the mandibular symphysis in *Ferrodraco* face dorsally. The mesiodistal lengths of the first two pairs of alveoli in *Aussiedraco* are greater than those of *Ferrodraco*. Although this might indicate that the anteriormost teeth of *Aussiedraco* were larger than those of *Ferrodraco*, it is possible that the *Aussiedraco* type specimen was slightly eroded prior to fossilisation, which would have exaggerated these features. Furthermore, if the suggestion that the first tooth pair in *Aussiedraco* was occupied by near-procumbent teeth is correct, then it differs from the condition in *Ferrodraco*, wherein the first tooth pair of the mandible are vertically oriented.

Perhaps most tellingly, two sulci were observed on the rostral tip of the mandible of *Aussiedraco* that were not present in *Ferrodraco*. Small sulci in this position on the mandible have not been reported in any other pterosaurs. However, it is possible that the *Aussiedraco* type specimen was slightly over-prepared (with acetic acid), in which case it is entirely possible that the two sulci might represent the bifurcating end of a mandibular groove. If this is correct, then this constitutes the second report of a bifurcating mandibular groove in anhanguerians. The first such report was made by Vila Nova *et al*.^34^ in their assessment of the *Cearadactylus atrox* holotype, which also lacks a mandibular crest^35^. This combination of characters, which has not been reported in any other pterosaurs, potentially represents a synapomorphy of *Aussiedraco* and *Cearadactylus*. However, *Cearadactylus atrox* differs from *Aussiedraco* in that it possesses a laterally expanded dentary.

#### Phylogenetic results

**Figure 7:**
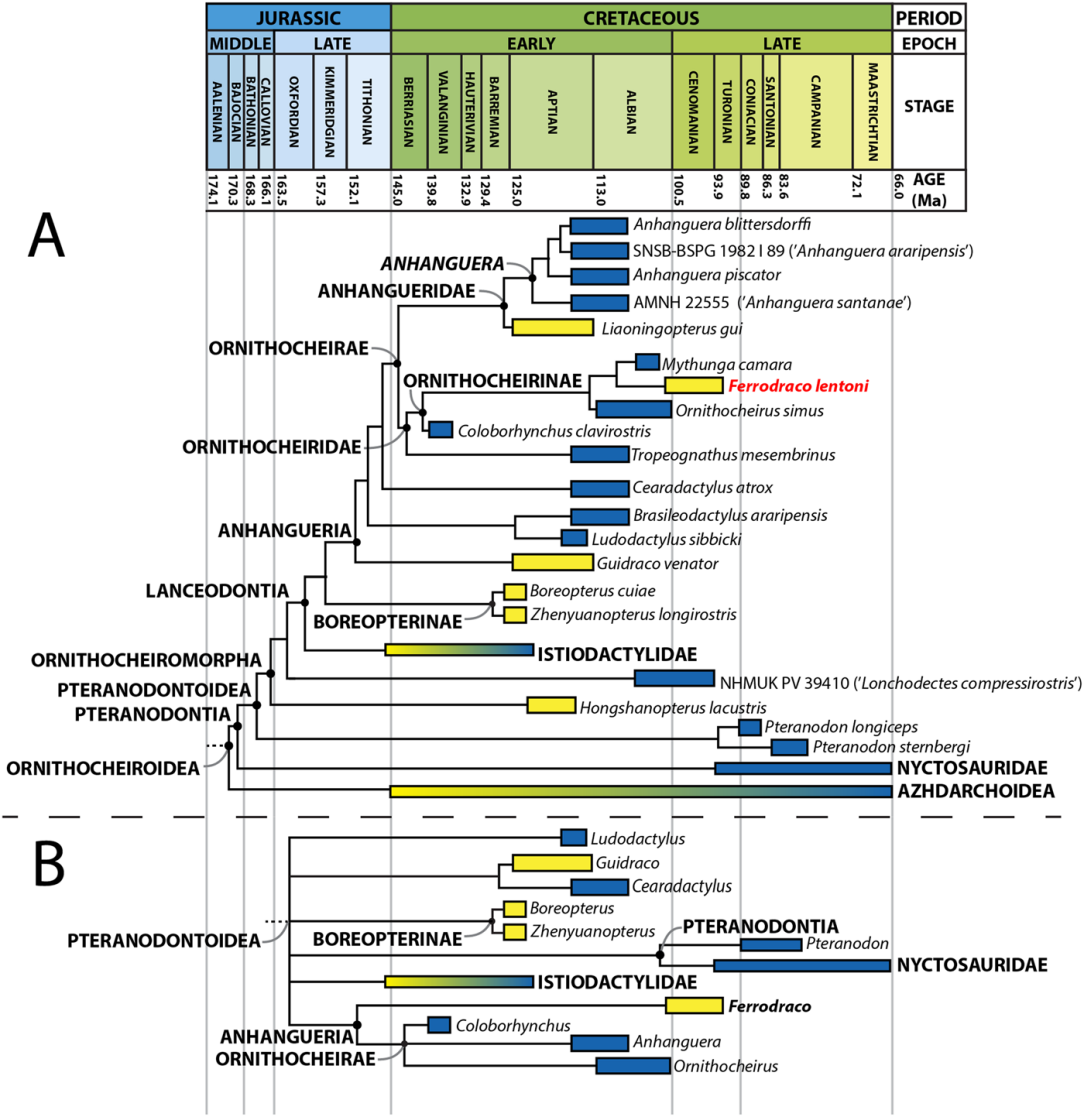
Time-calibrated phylogenetic trees of Ornithocheiroidea (Pterosauria: Pterodactyloidea), with some non-anhanguerian nodes collapsed for simplicity. The box next to each taxon demarcates its temporal range (including stratigraphic uncertainty), whereas the colour of each box reflects the palaeoenvironmental setting from which the taxon derives (yellow = terrestrial; blue = marine). (**A**) Tree based on the matrix of Andres *et al*.^19^, with *Ferrodraco lentoni* gen. et sp. nov. and *Mythunga camara* included; (**B**) Tree based on the matrix of Lü *et al*.^36^, with *Ferrodraco lentoni* gen. et sp. nov. included.

We included *Ferrodraco lentoni* in two phylogenetic analyses. In the first (modified from Pentland and Poropat^9^, which was in turn modified from Andres *et al*.^19^) *Ferrodraco* was resolved as the sister taxon to *Mythunga camara* (Fig. 7A). *Ornithocheirus simus* and *Coloborhynchus clavirostris* were resolved as the successive sister taxa to this clade within Ornithocheirinae, and *Tropeognathus mesembrinus* was resolved as sister taxon to Ornithocheirinae within Ornithocheiridae. In the second analysis (modified from Lü *et al*.^36^), *Ferrodraco* was resolved as the sister taxon to a polytomy (equivalent to Ornithocheirae *sensu* Andres *et al*.^19^) comprising *Anhanguera*, *Coloborhynchus* and *Ornithocheirus* (Fig. 7B); this clade fits the definition of Anhangueria *sensu* Andres *et al*.^19^.

## Discussion

**Figure 8:**
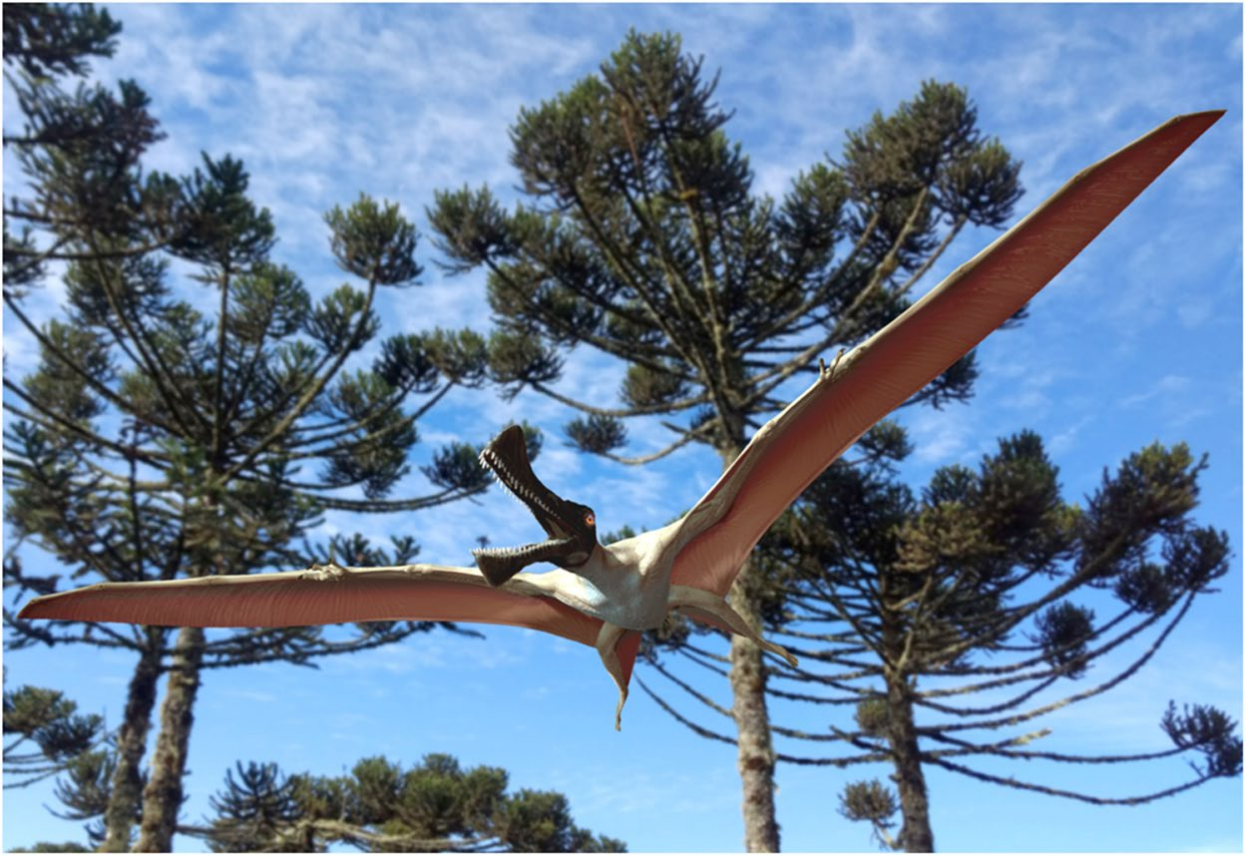
Life restoration of *Ferrodraco lentoni* gen. et sp. nov. as an ornithocheirid pterosaur. Illustration by T.R.T.

Phylogenetic analysis indicates that *Ferrodraco* (Fig. 8) and *Mythunga* belong to the Ornithocheirinae *sensu* Andres *et al*.^19^, with two pterosaurs from England — *Ornithocheirus simus* from the Albian Cambridge Greensand and *Coloborhynchus clavirostris* from the Valanginian Hastings Beds — as successive sister taxa within that clade. The only non-ornithocheirine ornithocheirid in our analysis was *Tropeognathus mesembrinus* from the Albian Romualdo Formation of Brazil. Given that the Australian pterosaurs are more closely related to taxa from England than to forms from Brazil, it would seem that ornithocheirids were cosmopolitan during the Early–mid-Cretaceous, which contrasts with the Gondwanan provincialism shown in several terrestrial vertebrate clades during that time^37–40^. Although this result might be viewed as somewhat surprising, given the incompleteness of the English material, this result is consistent with previous interpretations that ornithocheirids could easily disperse across oceanic barriers^41–43^.

Although our results do not support a specific biogeographic signal, many late Early Cretaceous pterosaur faunas that include anhanguerians also feature azhdarchoids. This includes the Barremian–Aptian Jehol Biota (summarised by Zhou^44^), the Aptian–Albian limestones of the Araripe Basin (summarised by Unwin and Martill^42^ and Leal *et al*.^45^), and the Albian–lower Cenomanian Kem Kem Beds (summarised by Jacobs *et al*.^46^). At present, *Ornithostoma sedgwicki*^47^ is the only edentulous pterosaur reported from the Cambridge Greensand; nevertheless, this consistent biogeographical pattern suggests that future excavations in the Winton Formation might also result in the discovery of azhdarchoids.

It has been suggested that anhanguerians went extinct at the end of the Cenomanian, based on their apparent absence from post-Cenomanian strata^43^. Their apparent absence follows a period of major environmental disturbance characterised by an increase in atmospheric and oceanic surface temperatures^48–50^, an increase in atmospheric carbon dioxide^51,52^, a global oceanic anoxic event^52–54^, and marine transgression^49,55,56^. At present, the majority of anhanguerians are known from restricted lagoon environments, with stable isotopic analysis of isolated teeth derived from the Araripe Basin indicating a piscivorous diet comprising both freshwater and marine fish^57^. Given that marine vertebrates and invertebrates were most severely impacted during the Cenomanian–Turonian event^58^, we suggest that anhanguerians were also impacted by a disruption in trophic interactions. However, if the teeth sampled from the Araripe Basin are not diagenetically altered and are indicative of the feeding behaviours of this clade more broadly, it is possible that anhanguerians in terrestrial settings might have persisted beyond the Cenomanian.

The presence of an anhanguerian in the Winton Formation is not surprising, given that several pterosaurs referred to the Ornithocheiridae have been reported from the underlying upper Albian Toolebuc and Mackunda formations. However, recent analyses of detrital zircons obtained from localities in the Winton area by Tucker *et al*.^20,21^ suggest that deposition of the northern part of the Winton Formation — from which *Ferrodraco lentoni* derives — might have taken place as late as the early Turonian. Given that *Ferrodraco* derives from a locality northeast of Winton, it potentially represents a late-surviving member of the Ornithocheiridae specifically, and of Anhangueria more broadly. Another possible anhanguerian from the post-Cenomanian of Australia is represented by a jaw fragment bearing two raised alveoli (WAM 68.5.11), derived from the Molecap Greensand and described by Kear *et al*.^12^. The jaw fragment was tentatively assigned by those authors to the Ornithocheiridae (*sensu* Unwin^17^) or Anhangueridae (*sensu* Kellner^23^). As noted by Kear *et al*.^12^, the Molecap Greensand preserves pollen and dinocysts that vary in age from the Cenomanian through to Coniacian. Given that both the Molecap Greensand and the *Ferrodraco* type locality lack refined age constraints, considerable further work is required before it can be determined whether Australia was a refugium for anhanguerian pterosaurs during the Late Cretaceous. At the very least, however, a strong case can be made for their persistence until the end-Cenomanian.

## Methods

### CT scan analysis

The *Ferrodraco lentoni* holotype specimen, AODF 876, was CT scanned at St Vincent’s Hospital Melbourne (Victoria, Australia) using a Revolution CT scanner (GE Medical Systems). The specimen was scanned at 140 kV and 401.5 mA, obtaining 0.625 cm voxel size. The CT data was imported into Mimics 21 (Materialise, Belgium) to enable visualisation of internal features.

### Nomenclatural acts

This published work and the nomenclatural acts it contains have been registered in ZooBank, the proposed online registration system for the International Code of Zoological Nomenclature. The ZooBank Life Science Identifiers (LSIDs) can be resolved and the associated information viewed by appending the LSIDs to the prefix http://zoobank.org/. The LSID for this publication is urn:lsid:zoobank.org:pub:5FC8D723-2340-4331-BD3A-87A3F5A95C80, and the LSIDs for the new erected groups and taxa are: urn:lsid:zoobank.org:act:435795CA-3D70-4895-98AD-AA277351C0E2 (*Ferrodraco*), and urn:lsid:zoobank.org:act:9A96B460-92A9-4259-AF2D-9A7C9FB6E75A (*Ferrodraco lentoni*).

### Phylogenetic analysis

In order to constrain the phylogenetic position of *Ferrodraco lentoni*, we scored it for two phylogenetic datasets. The first was the modified version of the Andres *et al*.^19^ dataset employed by Pentland and Poropat^9^ to constrain the phylogenetic position of *Mythunga camara*; the second was that of Lü *et al*.^36^, for which we scored both *Ferrodraco lentoni* and *Mythunga camara*. In the latter dataset, we also changed the scores of characters 51–65 from ‘0’ to ‘−’ for all edentulous taxa (i.e. *Nyctosaurus*, *Muzquizopteryx*, *Pteranodon*, *Tapejara*, *Tupandactylus*, *Sinopterus*, *Huaxiapterus*, *Shenzhoupterus*, *Chaoyangopterus*, *Tupuxuara*, *Thalassodromeus*, *Quetzalcoatlus*, *Zhejiangopterus* and *Azhdarcho*); however, this had no discernible effect on the outcome.

Analyses of both datasets were conducted in TNT v. 1.5 (Tree analysis using New Technology)^59^. We analysed the modified Andres *et al*.^19^ matrix following the methodology used by the original authors. This resulted in a single most parsimonious tree (MPT) of 866.272 steps, which was almost completely resolved (except for the three taxa within Nyctosauridae, which formed a polytomy as in the original analysis). The inclusion of *Ferrodraco lentoni* stabilised the position of *Mythunga camara* within Anhangueria, such that this clade was fully resolved in this analysis (unlike in Pentland and Poropat^9^, wherein much of Anhangueria lacked resolution). We also analysed the modified Lü *et al*.^36^ following the methodology used by the original authors, although the inclusion of *Mythunga camara* proved sufficiently problematic that it was pruned. Analysis of the pruned matrix resulted in 31,116 MPTs of 517 steps. The topology of the strict consensus tree obtained from these 31,116 MPTs was almost identical to that of the original tree presented by Lü *et al*.^36^. However, in our tree Pteranodontia was slightly better resolved, with *Elanodactylus*, *Beipiaopterus*, *Feilongus* and *Moganopterus* basal to other pteranodontians.

## Supplementary information

Dataset 1

Supplementary Information
