# Supplementary material for: Ferrodraco lentoni gen. et sp. nov., a new ornithocheirid pterosaur from the Winton Formation (Cenomanian–lower Turonian) of Queensland, Australia
Source: Sci Rep. 2019 Oct 3;9:13454. doi: 10.1038/s41598-019-49789-4 (PMC6776501; doi:10.1038/s41598-019-49789-4)
Supplement: Supplementary file 2 — Supplementary Information [file 41598_2019_49789_MOESM2_ESM.docx]

***Ferrodraco lentoni* gen. et sp. nov., a new ornithocheirid pterosaur from the Winton Formation (Cenomanian–lower Turonian) of Queensland, Australia**

**Adele H. Pentland, Stephen F. Poropat, Travis R. Tischler, Trish Sloan, Robert A. Elliott, Harry A. Elliott, Judy A. Elliott & David A. Elliott

Supplementary Information**

Table S1. Isolated teeth of *Ferrodraco lentoni* (mm)

| **Tooth number** | **Apicobasal height (mm)** | **Mesiodistal width (mm)** | **Height:Width Ratio** | **Notes** |
| --- | --- | --- | --- | --- |
| A | 11 (pres.) | 4 (pres.) | 2.8* | Incomplete at crown and base |
| B | 15 | 2 | 7.5 | Slender tooth, constricted at base comprising tooth root |
| C | 7 (pres.) | 2 (pres.) | 3.5* | Incomplete apicobasally, at crown and at base |
| D | 9 (pres.) | 3 (pres.) | 3* | Incomplete apicobasally revealing pulp cavity, incomplete at base |
| E |  |  |  |  |
| F | 29 | 7 | 4.1 | Comprising two pieces, pulp cavity infilled with ironstone, tooth root not preserved |
| G | 10 (pres.) | 4 (pres.) | 2.6* | Incomplete at crown and base |
| H | 8 (pres.) | 3 (pres.) | 2.7* | Incomplete at crown and base |
| I | 19 | 4 | 4.8 | Near complete tooth, constricted at base |
| J | 9 (pres.) | 3 (pres.) | 3* | Incomplete apicobasally, at crown and at base |
| K | 16 (pres.) | 4 | 4* | Incomplete at crown and base |
| L | 21 (pres.) | 5 | 4.2 | Incomplete at base |
| M | 23 | 4 | 5.8 | Tooth crown slightly worn, constricted at base with tooth root |
| N | 19 | 3 | 6.3 | Comprising two pieces, tooth joins the mandible |
| O | 22 | 4 | 5.5 | Complete tooth comprising tooth root |
| P | 8 (pres.) | 4 | 2* | Incomplete at base |
| Q | 10 (pres.) | 3 (pres.) | 3.3 | Incomplete apicobasally, at crown and at base |
| R | 29 (pres.) | 6 | 4.8 | Comprising two pieces; base found at surface by B. Elliott, tooth crown discovered during excavation; tooth crown broken |
| S |  |  |  |  |
| T | 15 | 4 | 3.8 | Near complete tooth, constricted at base, preserves tooth root |
| U | 13 (pres.) | 4 | 3.3* | Incomplete at base |
| V | 12 (pres.) | 4 | 3* | Incomplete at base |
| W | 16 | 3 | 5.3 | Comprising two pieces; broken apicobasally revealing pulp cavity; constricted at base |
| X | 9 (pres.) | 4 (pres.) | 2.3* | Broken at tooth crown and base |
| Y | 12 | 2 | 6 | Constricted at base, preserves tooth root |
| Z |  |  |  |  |
| AA | 12 (pres.) | 3 (pres.) | 4* | Incomplete apicobasally, at crown and at base |
| AB |  |  |  |  |
| AC | 18 | 4 | 4.5 | Base constricted, partially preserves tooth root |
| AD | 22 (pres.) | 3 | 7.3* | Incomplete at base |
| AE | 10 | 3 | 3.3 | Near complete, constricted at base, lingually recurved |
| AF | 10 (pres.) | 4 (pres.) | 2.5* | Broken at tooth crown, incomplete at base |
| AG | 15 (pres.) | 6 (pres.) | 2.5* | Comprising two separate pieces; incomplete apicobasally, at tooth crown and base |
| AH | 10 (pres.) | 4 | 2.5* | Worn tooth crown, broken at base |
| AI | 11 (pres.) | 3 | 3.7* | Incomplete at base |
| AJ | 9 (pres.) | 3 (pres.) | 3* | Incomplete at tooth crown and base |
| AK | 9 (pres.) | 4 (pres.) | 2.3* | Comprising two pieces, incomplete at tooth crown and base |
| AL | 10 (pres.) | 4 | 2.5* | Incomplete apicobasally, at tooth crown and base |
| AM | 9 (pres.) | 3 | 3* | Incomplete at tooth crown and base |
| AN |  |  |  |  |
| AO | 7 | 3 | 2.3 | Basally constricted indicating the tooth root is partially preserved |
| AP | 9 (pres.) | 2 (pres.) | 4.5* | Incomplete apicobasally, at tooth crown and base revealing pulp cavity |
| AQ | 7 (pres.) | 3 | 2.3* | Incomplete at base; lingual surface partially obscured by ironstone |
| AR | 9 (pres.) | 2 | 4.5* | Preserves only part of a lateral surface |
